# Molecular dynamics simulations revealed structural differences among WRKY domain-DNA interaction in barley (*Hordeum vulgare*)

**DOI:** 10.1186/s12864-018-4506-3

**Published:** 2018-02-12

**Authors:** Bharati Pandey, Abhinav Grover, Pradeep Sharma

**Affiliations:** 1 Plant Biotechnology Unit, ICAR-Indian Institute of Wheat and Barley Research, Karnal, 132001 India; 2 School of Biotechnology, Jawaharlal Nehru University, New Delhi, 110067 India; 3 Present address: Department of Biotechnology, Panjab University Chandigarh, Chandigarh, 160014 India

**Keywords:** WRKY, Molecular modeling, Molecular dynamics, HADDOCK, Barley

## Abstract

**Background:**

The WRKY transcription factors are a class of DNA-binding proteins involved in diverse plant processes play critical roles in response to abiotic and biotic stresses. Genome-wide divergence analysis of *WRKY* gene family in *Hordeum vulgare* provided a framework for molecular evolution and functional roles. So far, the crystal structure of WRKY from barley has not been resolved; moreover, knowledge of the three-dimensional structure of WRKY domain is pre-requisites for exploring the protein-DNA recognition mechanisms. Homology modelling based approach was used to generate structures for WRKY DNA binding domain (DBD) and its variants using *At*WRKY1 as a template. Finally, the stability and conformational changes of the generated model in unbound and bound form was examined through atomistic molecular dynamics (MD) simulations for 100 ns time period.

**Results:**

In this study, we investigated the comparative binding pattern of WRKY domain and its variants with W-box *cis*-regulatory element using molecular docking and dynamics (MD) simulations assays. The atomic insight into WRKY domain exhibited significant variation in the intermolecular hydrogen bonding pattern, leading to the structural anomalies in the variant type and differences in the DNA-binding specificities. Based on the MD analysis, residual contribution and interaction contour, wild-type WRKY (HvWRKY46) were found to interact with DNA through highly conserved heptapeptide in the pre- and post-MD simulated complexes, whereas heptapeptide interaction with DNA was missing in variants (I and II) in post-MD complexes. Consequently, through principal component analysis, wild-type WRKY was also found to be more stable by obscuring a reduced conformational space than the variant I (HvWRKY34). Lastly, high binding free energy for wild-type and variant II allowed us to conclude that wild-type WRKY-DNA complex was more stable relative to variants I.

**Conclusions:**

The results of our study revealed complete dynamic and structural information about WRKY domain-DNA interactions. However, no structure base information reported to date for WRKY variants and their mechanism of interaction with DNA. Our findings highlighted the importance of selecting a sequence to generate newer transgenic plants that would be increasingly tolerance to stress conditions.

**Electronic supplementary material:**

The online version of this article (10.1186/s12864-018-4506-3) contains supplementary material, which is available to authorized users.

## Background

Barley (*Hordeum vulgare* L.) is amongst the world’s earliest domesticated and most important cereal crops. It is diploid in nature with a large genome of 5.1 Gb [1]. Barley crop growth, development and crop yield are limited by unfavorable conditions and factors such as water stress salinity and extreme temperatures [2]. Several transcription factor families have been shown to be involved in the defense against these adverse stress conditions [3]. The WRKY family is among them and play key roles in modulating gene expression during defense in response to biotic and abiotic stress [4]. The first *WRKY* gene (*SPF1*) was identified in sweet potato [4], since then it has been identified in various plant species [5–13].

*WRKY* gene family is one of the largest and extensively studied transcription factor gene families across the plant kingdom. WRKY proteins are described by the presence of highly conserved WRKY DNA binding domain (DBD) and a unique C2H2 zinc finger motif [14]. The core sequence or DNA binding sequence of the WRKY protein is WRKYGQK with some frequently occurring variants in crop plants. In rice, the WRKY family members have 19 variants of the WRKY domain where WRKYGEK (seven) and WRKYGKK are the two common variants shared by seven and five domains respectively [5]. Okay et al. [2014] also identified 13 different WRKY motifs (WRKYGQK, WRKYGEK, WRKYGQE, WLKYGKK, LRKYGPK, WRNYGQN, WRKYGQK, WRKDHQK, WSKYGQK, WTKYGQK, GRKYGEK and WMKYGQK) in wheat [6]. Similarly, in barley conserved WRKY domain had other forms such as WRKYGKK (*Hv*WRKY18, *Hv*WRKY19 and *Hv*WRKY20), WRKYGQN (*Hv*WRKY33, *Hv*WRKY34 and *Hv*WRKY36) and WRKYGQM (*Hv*WRKY24) [15]. WRKY proteins are classified into three groups based on the number of WRKY domain and the type of zinc finger-like motif. Those with two WRKY domains belong to group I while those with a single WRKY domain belong to group II and III were characterized [7]. The domain binds to the W-box DNA motif (TTGACT/C) which is located in the promoter region of downstream genes and regulates the signalling cascade. WRKY proteins have been involved in modulating gene expression in defense against pathogens, plant growth and development, senescence, biosynthesis and hormonal regulation, drought, cold and salt [5, 8–11]. In the physiological processes, *WRKY* genes are regulated by phosphorylation through Mitogen-activated protein kinases (MAPKs). WRKY74 from *Oryza sativa* regulates Pi homeostasis, Fe starvation and cold stress in rice [12]*. WRKY71* from *Arabidopis thaliana* (At) regulates shoot branching by activating *RAX* genes and on the other hand escalates flowering by regulating FLOWERING LOCUST and LEAFY genes [13]. *WRKY46* from *A. thaliana* regulates facilitating the growth of lateral roots in osmotic/salt stress through modulation of ABA signalling and auxin homeostasis [16].

Protein-DNA interactions play an important role in the translation of genomic information to the biological significances. Since recognition of specific DNA sequences by proteins is very complex, it is difficult to predict how those proteins interact with DNA by experimental approaches. Therefore, use of time and cost-effective computational techniques such molecular dynamics (MD) simulations, the docking study are required at this juncture to speed up the process of knowledge recovery and to narrow down the search space for experimental protocols. Complex crystal structure of WRKY domain and W-box DNA from *Arabidopsis thaliana At*WRKY1 protein was solved using NMR method (2LEX and 2LEX) [17]. Recently in *Arabidopsis*, variation in DNA-binding specificities in different *At*WRKY groups was studied using 10 ns molecular dynamics and in vitro experiments [18]. However, no such studies, understanding its structural framework for the DNA recognition mechanism, were available in barley.

In the present study, we constructed a homology- based WRKY protein model and comparative MD simulations of barley WRKY and its variants in order to understand molecular mechanisms of WRKY TFs and how DNA binding regions of these TFs interact with DNA. The outcomes of this study may provide a platform for future studies regarding the function of *WRKY* genes in response to stress in barley*.*

## Methods

### Sequence analysis

Based on available literature survey, the most common occurring variants of WRKY DNA binding domain (DBD) in barley was selected for the study. The reviewed 66 amino acid sequence of HvWRKY46 (wild-type WRKY), HvWRKY34 (variant I; Q17E) and, HvWRKY19 (variant II; Q17K) with Q6VWJ6, B2KJ76, B2KJ62 UniProt ID were retrieved from UniProt database (www.uniprot.org). These were highly annotated and non-redundant protein sequence.

### Generation of structural models for protein and DNA

All WRKY variants showed > 40% identity with the template in the PSI-BLAST (https://blast.ncbi.nlm.nih.gov/Blast.cgi), was employed for constructing three dimensional (3D) protein structure using homology modeling approach. Homology models were constructed for WRKY DBD, using SWISS model server (https://swissmodel.expasy.org/). WRKY DBD mediates signalling through binding to the DNA sequence 5’-TTGACC-3′ (W-box). Three dimensional B-form of W-box was retrieved from PDB ID: 2LEX (Complex of the C-terminal WRKY domain of AtWRKY4 and a W-box DNA). The reliability of the generated protein model was verified using Structure Analysis and Verification Server version 4. (SAVES) [19]. The server integrates analysis from multiple widely-used validation algorithms (such as PROCHECK, ERRAT) taking into account certain geometrical parameters, or topological, to validate goodness-of-fit between model structure and experimental data.

### Docking protocol of the protein-DNA complexes

To investigate the WRKY DBD-DNA interactions, WRKY DBDs were docked into the specific site of DNA (W-box) using HADDOCK (High Ambiguity Driven protein-protein Docking) web server (version 2.2) as mention in our previous work [20, 21]. Position from 12 to 18 was designated as active residues from WRKY DBD for wild-type and variants. Passive residues were automatically defined around active residues. Based on active and passive residues Ambiguous Interaction Restraints (AIR) was generated. The illustration and visualization of the final docked complex were completed with UCSF Chimera [22].

### Molecular dynamics (MD) simulations of WRKY domain and its complexes

The wild-type and variants (I and II) WRKY domain were subjected to MD simulations using Gromacs 5.0 software package [23, 24]. For unbound and bound WRKY DBDs simulations AMBER99SB-ILDN protein, the nucleic AMBER94 force field was applied [25, 26]. All the systems were solvated in cubic water box using the minimal with Simple Point Charge (SPC) water model [27]. Ions were added to neutralize the entire system by substituting the water molecule ensuring overall charge neutrality of the wild-type and variants (I and II) WRKY DBD. At first, to remove the steric clash, steepest descent algorithm energy-minimized for 50,000 cycles was performed. Further minimized system was equilibrated into NVT and NPT phases for 1000 ps using a similar methodology as mentioned in our previous work [28–31]. Subsequently, temperature (300 K) and pressure (1 bar) of the system was maintained using Vrescale, a modified Berendsen thermostat temperature coupling method [5] and Parrinello-Rahman pressure coupling method [6] respectively. Finally, the well equilibrated systems were subjected to a production run at 300 K and 1 bar pressure for 100,000 ps. Dynamic behavior and stability of each residue of the wild-type and variants (I and II) WRKY DBD were also analyzed including root mean square deviation (RMSD), the radius of gyration (Rg), root mean square fluctuation (RMSF), solvent accessible surface area (SASA) and hydrogen bond profile using Gromacs inbuilt tools. Representative structures were extracted using RMSD conformational clustering algorithm, a gmx-cluster module of Gromacs. A cut-off of 2.0 Å was applied and the maximally occupied clusters were extracted by taking into account the protein conformation with the lowermost RMSD to the centroid. The schematic diagrams of protein-DNA interactions in pre- and post-MD simulated complex were deduced using Nucplot (https://www.ebi.ac.uk/thornton-srv/software/NUCPLOT/).

### Calculation of binding free energy

The binding free energies were calculated for WRKY-DNA complexes using the molecular mechanics/Poisson Boltzmann surface area (MM/PBSA) approach [32]. The analysis was performed using g_mmpbsa tool of Gromacs. Contribution of each residue towards binding free energy was calculated using MmPbSaDecomp.py python script.

### Essential dynamics study

Essential dynamics (ED) or Principal component analysis (PCA) is a statistical approach to decrease the complexity of data by retrieving collective motion of atoms in simulated trajectories that are significantly essential for the biological process and molecular function. Determination of eigenvectors and eigenvalues projected along the first two principal components were performed within Gromacs [33]. In the first step of ED, a covariance matrix was generated using from the equilibrated simulated time from the trajectory after elimination of the rotational and translational movements. The matrix was then diagonalized to identify a set of eigenvectors and eigenvalues. The, gmx-covar, gmx-anaeig and gmx-sham module of Gromacs was used to compute the PCA and Gibbs free energy landscapes in the PC1 vs PC2 conformational space [34].

## Results and discussion

### Sequence analysis and expression pattern

**Fig. 1:**
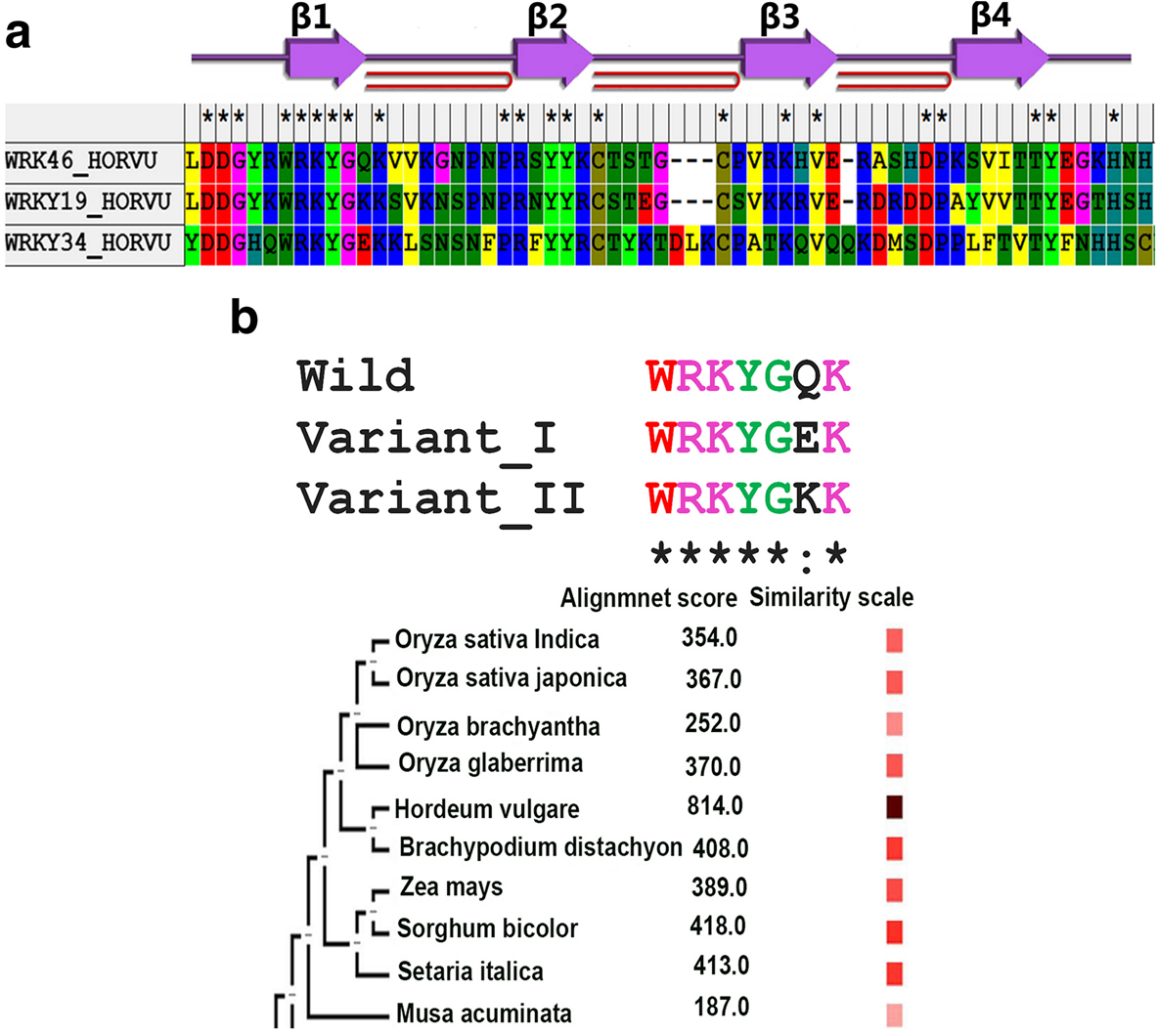
Sequence alignment of the WRKY domain (**a**) from HvWRKY46 (wild WRKY; group I), HvWRKY34 (variant I; group III) and HvWRKY19 (variant II; group II). Mutation of Q- > E and Q- > K at 17th position is represented double colon and black color residue (numbering according to start of WRKY domain) (**b**) Co-occurrence pattern of the WRKY family across the plant species

The WRKY domain is comprised of 60–70 amino acids residue long DBD characterized by a highly conserved heptapeptide WRKYGQK motif [35]. In this study, we chose *Hv*WRKY46 (termed as wild-type WRKY) a group I member, exhibiting two DBDs both at N- and C-terminals, but only C-terminal WRKY domain is responsible for sequence-specific binding to the DNA. The *Hv*WRKY34 from group III possess WRKYGEK motif, (where a substitution was observed from polar uncharged amino acid glutamine in wild-type to polar negatively charged aliphatic amino acid, glutamic acid; Q17E) was termed as variant I. Similarly, the *Hv*WRKY19 from group II, contains WRKYGKK motif (showing substitution from polar uncharged amino acid glutamine in wild- type to polar positively charged amino acid, lysine; Q17K) was termed as variant II (Fig. 1a). Based on these substitution, the respective sequences showed divergence in the molecular phylogenetic analysis of WRKY domain and were classified in different groups in barley [7, 15]. Previously, it was demonstrated that the substitution of any residue in WRKYGQK peptide into alanine, remarkably abolished the DNA binding activity [36]. In order examine the effect of substitutions on protein function, structural properties, and DNA binding pattern, extensive computational analysis has been carried out. The occurrence pattern of the *WRKY* gene family across the plant species was studied using STRING database. The results revealed that *Sorghum bicolor* as the closest homolog of barley *WRKY* gene with a high alignment score of 418.0 followed by *Setaria italica* and *Brachypodium distachyon* (alignment score: 413.0 and 408.0) respectively (Fig. 1b). The comparative analysis of physicochemical properties such as theoretical pI, Instability index (II), and Aliphatic index (AI) for the wild-type, variant I and variant II WRKY domain was calculated using Protparam as detailed in Table 1.

**Table 1:** Physicochemical Parameters computed using Expasy’s ProtParam tool

WRKY	Accession No.	Length	M. wt(kDa)	pI	EC	II	AI	GRAVY
Wild-type	AAQ63880.1	58	6746.57	9.74	13,075	28.50	40.17	−1.376
Variant I	ABI13400.1	58	6842.56	9.39	14,565	18.30	33.45	−1.543
Variant II	ABI13385.1	62	7514.43	9.08	14,565	43.58	29.84	−1.231

*EC* Extinction coefficients, *II* Instability index, *AI* Aliphatic index, *GRAVY* Grand average of hydropathicity

### Construction of protein structure

To build the 3D structure of all WRKY DBDs, we extracted the WRKY DBD (60–70 amino acids residue) from full-length WRKY protein. PSI-BLAST predicted chain A of solution structure of the C-terminal WRKY domain of *A. thaliana* (Atwrky4) (PDB ID: 1WJ2_A) were found as best match template for wild-type WRKY DBD and variant II with 83% and 61% sequence identity, 100% query coverage and e-value of 4e-38 and 2e-26 respectively [Additional file 1: Figure S1 (a and b)]. Whereas, chain B of the crystal structure of PopP2 in complex with IP6, AcCoA and the WRKY domain of RRS1-R (PDBID: 5W3X) was predicted to be the template for a variant I with 49% sequence identity, 78% query coverage and 2e-13 e-value [Additional file 1: Figure S1 (c)]. Protein templates selected through BlastP results were used for generating protein models in Swiss model program. The modelled WRKY DBD comprised of four-stranded anti-parallel β-sheets with a zinc ion held at a place by two cysteines and two histidines in tetrahedral coordination geometry. It was also reported that Zn ion of TFIIA protein was essential for maintaining the correct protein conformation for sequence-specific binding to DNA [37].

### Validation of 3D protein structures

Finally, the generated homology models for WRKY DBDs (wild-type and variants) were checked for an overall model quality prior to molecular docking. Ramachandran plot generated by PROCHECK gives information about the backbone dihedral angels Phi against Psi distribution of the amino acid residues in the protein structure [38]. The Ramachandran plot obtained for the wild-type, variants (I and II) WRKY DBD showed that 85.1%, 84.2% and 82.5% residues were found in most favored region respectively. Whereas, 16.4% residues of wild-type, 14.0% of variant I and 17.5% of variant II were present in the additional allowed regions signified that the constructed models were accurate and trustworthy for further docking and molecular dynamics (MD) experiments (Table 2). The models were also checked for its fold reliability using ProSA-web server that predicts energy profile in terms of Z score [39]. The predicted Z scores were − 3.1, − 2.93 and − 3.34 for the wild-type, variant I and variant II WRKY DBDs respectively, indicating the accuracy of the models. The backbone conformation and non-bonded interactions within a cut-off distance of 3.5 Å between different pairs of atom types (CC, CN, CO, NN, NO, OO) were measured for each generated models by the ERRAT plot [40]. The overall quality factor for wild-type, variant I and II were 89.79, 87.71 and 76.78, respectively, confirmed the correctness of the generated models.

**Table 2:** Ramachandran plot statistics and scores for WRKY DBD variants

WRKY protein		Most favored regions (%)	Additional allowed regions (%)	Generously allowed region (%)	Disallowed region (%)
Wild-type	Pre-simulation	81.8	16.4	1.8	0.0
	Post-simulation	85.5	14.5	0.0	0.0
Variant I	Pre-simulation	84.2	14.0	0.0	1.8
	Post-simulation	84.2	14.0	1.8	0.0
Variant II	Pre-simulation	82.5	17.5	0.0.	0.0
	Post-simulation	89.5	10.5	0.0.	0.0

### Molecular dynamics simulations of the modeled protein

**Fig. 2:**
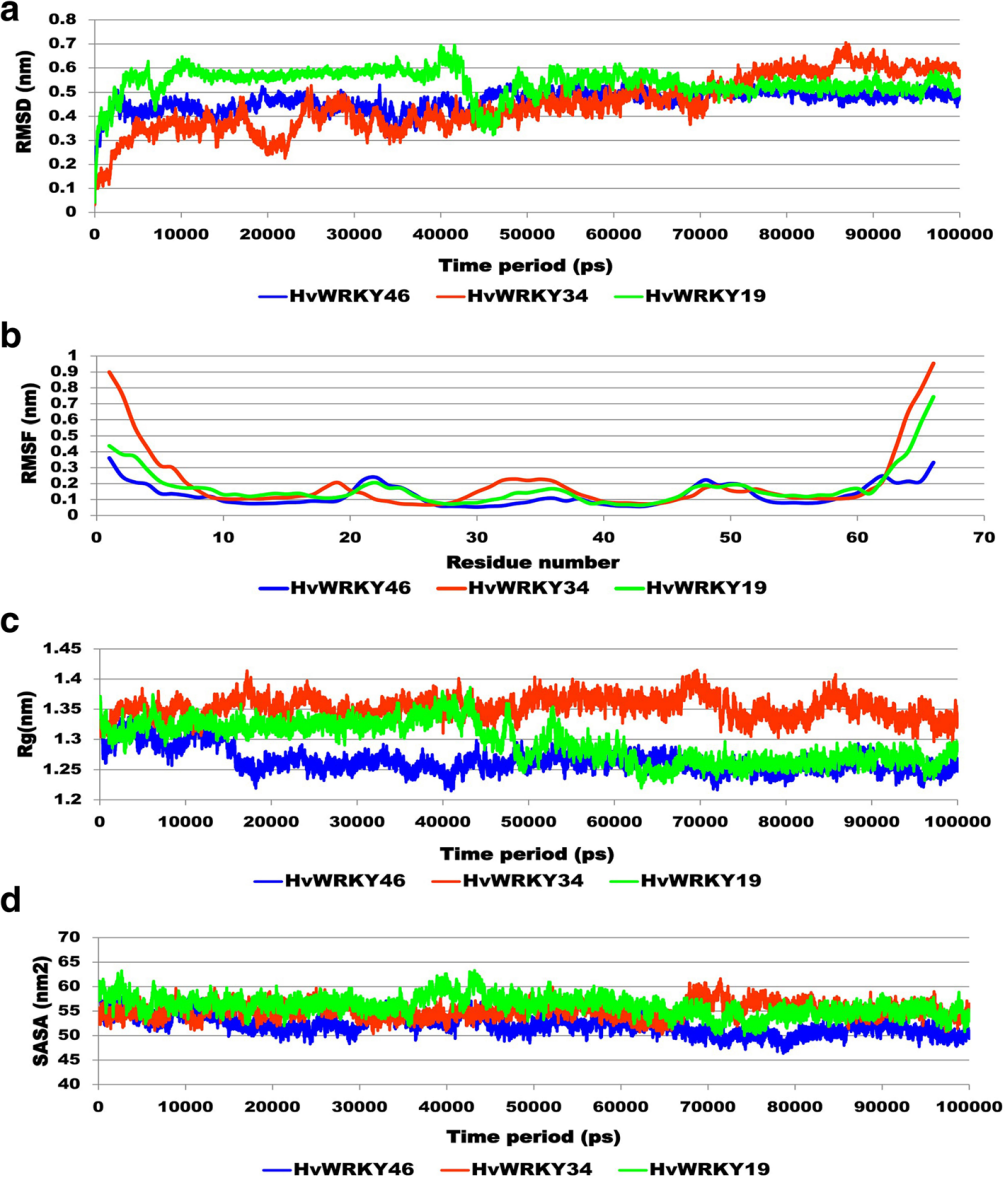
Comparative plots were of (**a**) RMSD was computed through least square fitting of backbone atom (**b**) RMSF (**c**) Radius of gyration and (**d**) Solvent accessible surface area (SASA) of wild-type and variants were generated from 100 ns MD simulations trajectory to investigate stability and fluctuation of WRKY DBD

To examine the change in the protein dynamics and stability, wild and variants WRKY DBDs models were subjected to 100 ns MD simulations. It observed that for wild-type WRKY and variants II remained stable during the entire simulation run with RMSD value ranging from 0.3 nm to 0.4 nm and 0.4 nm to 0.5 nm respectively. While, variants II was characterized by higher continuous RMSD fluctuations till 90,000 ps, thereby resulting in average backbone RMSD of 0.7 nm respectively, which was higher as compared to wild-type (Fig. 2a). Lower RMSD value of the wild-type indicated its stability and provided a suitable basis for further analysis.

The dynamic behaviour of individual amino acid residues for wild-type and variants (I and II) WRKY domain was determined in terms of RMSF values, denoted by the peak elevation. RMSF plot indicated similar residue fluctuation profile for wild-type and variant I with an average RMSF of 0.12 nm and 0.17 nm respectively (Fig. 2b). The maximum fluctuation was seen at 20–30 positions. The residues involved in DNA binding namely, Trp12, Arg13, Lys14, Tyr15, Gly16, Glu17, and Lys18 exhibited lower fluctuations with RMSF value indicating more stability with a pronounced role these residues in interaction (Table 3). RMSF plot for the variant I was characterized by higher continuous fluctuations, indicating that substitution had an effect on the flexibility of the protein variants throughout the simulations. For variant I maximum fluctuation occurred in the residue ranged from 30 to 40 position. However, critical residues (Trp12, Arg13, Lys14, Tyr15, Gly16, Gln17, and Lys18) involved in DNA binding were found to be quite high for variant I as compared to wild-type and variant II. The radius of gyration (Rg) indicates compactness of protein. Rg plot for alpha (α) carbon atoms of protein vs time at 300 K is described in Fig. 2c. The average Rg score for the wild-type, variant I and variant II were found to be 1.26 nm, 1.35 nm and 1.30 nm respectively. The graph indicated a simultaneous decrease in globularity for the variants I and II with an increase in the Rg score throughout the simulation (Fig. 2c). Rg results indicated that variants (I and II) had least compactness of its structure, whereas the wild-type WRKY DBD was highly compact. This also confirms that point mutation in the conserved site caused structural destabilizing effects leading to the loss of protein compactness in the WRKY variants (I and II).

**Table 3:** RMSF profile of WRKY DBD for WRKY DBD variants

WRKY	Trp12	Arg13	Lys14	Tyr15	Gly16	Glu17/Gln17/Lys17	Lys18
Wild-type	0.0751	0.0751	0.0765	0.0818	0.0833	0.0908	0.0883
Variant I	0.1045	0.1045	0.1112	0.1123	0.1219	0.1303	0.1745
Variant II	0.1278	0.1278	0.1377	0.1343	0.1383	0.1188	0.109

Solvent accessible surface area (SASA) for WRKY DBD was computed with respect to time as depicted in Fig. 2d. It was observed that the wild-type exhibited average SASA value of 52.12 nm^2^ whereas variant I and variant II were found to reveal the high SASA value of 55.06 nm^2^ and 56.08 nm^2^ respectively, which signified a greater magnitude of flexibility and instability (Fig. 2d). Long variation in the Rg and SASA plot of variant II indicated foremost structural changes which might induce a significant decrease in the occupancy of the most hydrogen bonds.

**Fig. 3:**
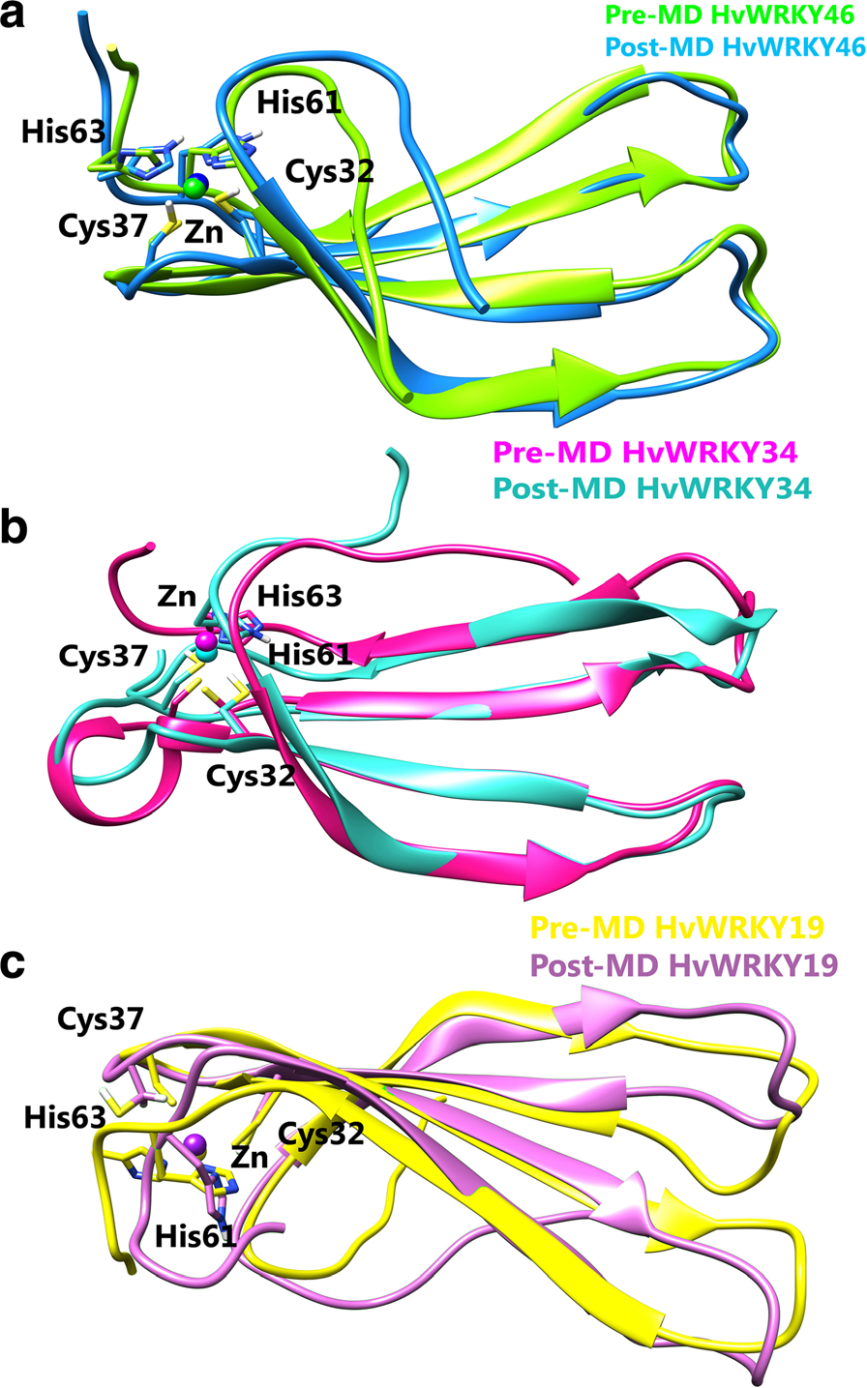
Ribbon representation showing superimposition of the pre- and post-MD models for (**a**) wild-type WRKY DBD (**b**) variant I (**c**) variant II

To investigate the conformation heterogeneity in the protein structure generated by MD simulations, clustering analysis was performed. Wild-type, variant I and II WRKY DBD conformations were distributed into 5, 15 and 18 clusters, respectively. The confirmation of top five most-populated clusters is shown in Additional file 2: Figure S2 (a-c). The cluster one comprised of 98.41%, 51.57% and 63.08% in wild-type, variant I and variant II with the average RMSD of 0.09 nm, 0.24 nm and 0.25 nm respectively. Pre- and post-MD simulated models were superimposed to analyze the similarity between the atomic coordinates (Fig. 3a-c). The RMSD value for wild- type, variant I and variant II was found to be 1.12 Å, 0.80 Å and 1.21 Å respectively. Secondary structure analysis was also carried out for wild-type and variant I and variant II (Table 4; Additional file 3: Figure S3). It was observed that β- Sheet remained consistent for wild and variants (I and II) and coil was found to decrease in HvWRKY34 and turn was observed to increase in HvWRKY16 as compared to the wild-type. Ramachandran plot analysis of the post-MD simulated structure of wild-type, variants (I and II) revealed that more residues located in the most favored regions as compared to pre-MD structures, suggested that simulated protein model is reliable for docking and further complex dynamics studies (Table 2).

**Table 4:** Comparative secondary structure analysis of WRKY DBD variants

Systems	Coil (C)%	β- Sheet (E)%	Turn (T)%
Wild-type	34.85	37.88	27.27
Variant I	27.27	37.88	34.85
Variant II	34.85	37.88	27.27

### Molecular docking analysis for wild-type WRKY DBD

**Fig. 4:**
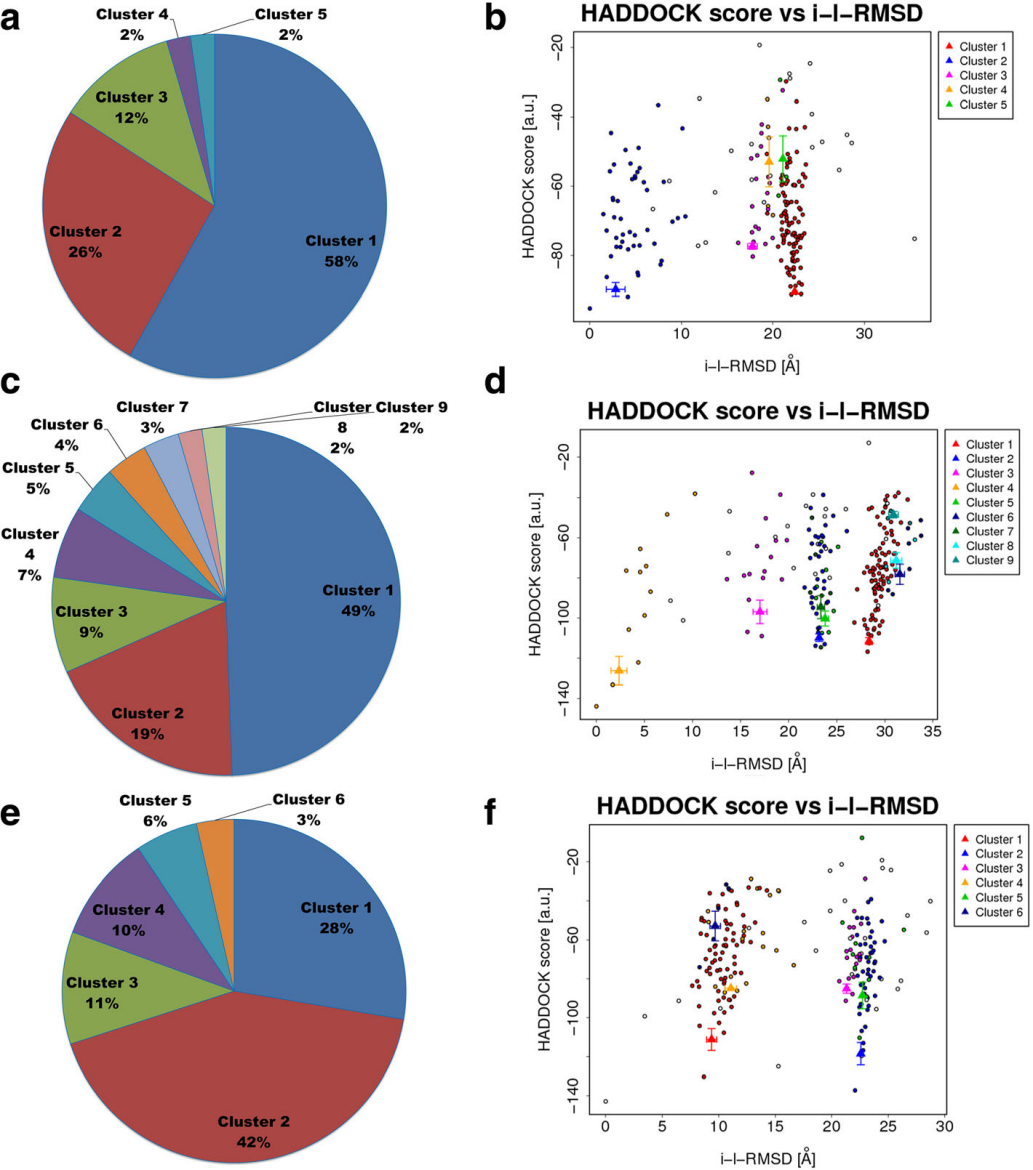
Pie-chart showing the distribution of Haddock clusters with cluster size, (**a**) wild-type WRKY-DNA docked complex (**c**) Variant I-DNA docked complex (**e**) Variant II-DNA docked complex (**b**) The HADDOCK scores of docked models were plotted against their i-RMSDs for wild-type WRKY-DNA docked complex (**d**) Variant I-DNA docked complex (**f**) Variant II-DNA docked complex. Color codes represent the i-RMSD values of all HADDOCK clusters

Molecular docking is one of the trustworthy approaches in structural biology used to explore the interacting residues between two molecules. The representative structure extracted using clustering algorithm was subjected to docking against DNA *cis-* regulatory motif (W-box). HADDOCK web server generated 5, 9 and 6 clusters for wild and variant I and variant II respectively. Size of cluster 1 was the largest for wild-type (58%) and variant I (49%) whereas cluster 2 was observed to have a large size for variant II (42%) [Fig. 4a–e]. Clusters were sorted based on the HADDOCK score; among all clusters the best scoring complex was selected based on highest HADDOCK score for plotting protein-DNA interaction. The HADDOCK score is a weighted sum of van der Waals, electrostatic, desolvation and restraint violation energies whereas, Z-score indicates the reliability of the selected complexes from cluster [41]. For each protein model, HADDOCK score vs i-lRMSD plot was generated. iRMSD (interface RMSD) calculated on the backbone (CA,C,N,O,P) atoms of all residues involved in intermolecular contact using a 10 Å cutoff. lRMSD (ligand RMSD) calculated on the backbone atoms (CA,C,N,O,P) of all (*N* > 1) molecules after fitting on the backbone atoms of the first (*N* = 1) molecule [Fig. 4b–f].

Electrostatic potential molecular surfaces provide insight into the molecular properties of the molecule. Additional file 4: Figure S4 (a) showed blue color on the surface of WRKY DBD, corresponds to the electrostatic surface potential at a particular point on the surface was positive charge and which complements the negatively charged DNA double helix might indicate a possible interaction site as shown in Additional file 4: Figure S4 (b).

**Fig. 5:**
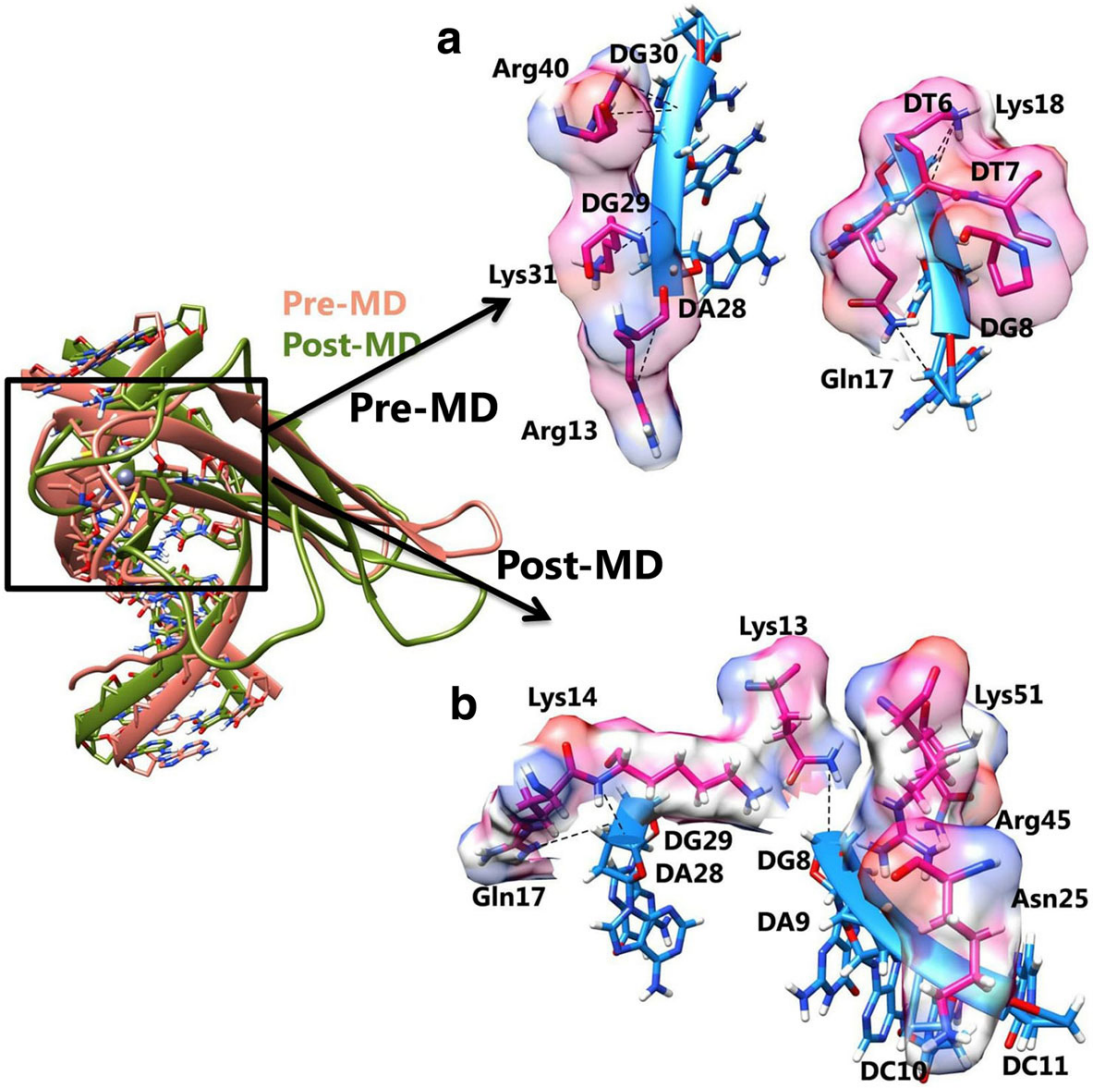
Interaction between wild-type WRKY DBD and DNA illustrating specific binding sites in (**a**) pre and (**b**) post-MD simulations

For the wild-type WRKY DBD, cluster 1 was selected as best docked complex based on highest HADDOCK score (− 90.5 ± 0.9) and Z-Score (− 1.1) (Table 5). In the WRKY-DNA complex, single Zinc atom was coordinated by Cys32, Cys37 (C2), His61 and His63 (H2) respectively. WRKY DBD binds to the major groove of DNA through highly conserve β1 strand of the β sheet (β1) as signature WRKYGQ (/K/E/) K motif lies within the β1 strand of domain. The wild WRKY-DNA complex was stabilized through the formation of seven hydrogen bonds (H-bond) formed by residues Arg13, Gln17, Lys18, Lys31 and Arg40 along with two hydrophobic interactions (Val19 and Pro26) (Table 8; Fig. 5a).

**Table 5:** Protein-DNA complexes generated for wild-type WRKY DBD with DNA motif using HADDOCK server

Models	HADDOCK score (a.u)	RMSD (A°)	Van der Waals energy (Kcal/mol)	Electrostatic energy (J)	Desolvation energy (kcal/mol	Restraints violation energy(kcal/mol)	Buried Surface Area (Å^2^)	Z-Score
**Cluster 1**	**−90.5 ± 0.9**	**14.8 ± 0.2**	**−35.6 ± 3.8**	**− 349.8 ± 21.3**	**11.9 ± 2.3**	**30.5 ± 29.20**	**1056.7 ± 63.5**	**−1.1**
Cluster 2	−89.8 ± 4.0	1.3 ± 0.9	−32.7 ± 4.8	− 348.4 ± 23.3	11.1 ± 2.3	14.4 ± 16.81	900.6 ± 115.4	− 1.0
Cluster 3	−77.4 ± 1.7	11.1 ± 0.9	−20.6 ± 3.1	− 361.5 ± 8.7	13.9 ± 2.9	16.6 ± 7.92	753.2 ± 71.2	−0.3
Cluster 4	−53.0 ± 14.4	8.8 ± 0.1	−29.6 ± 7.0	− 192.6 ± 32.9	13.0 ± 4.6	21.6 ± 22.34	971.4 ± 204.7	1.2
Cluster 5	−52.1 ± 13.3	13.8 ± 0.4	−21.9 ± 3.8	− 234.6 ± 62.1	11.4 ± 3.7	53.0 ± 32.58	625.3 ± 135.8	1.2

Bold cluster selected for further analysis

### Molecular docking analysis for variant I and II

**Fig. 6:**
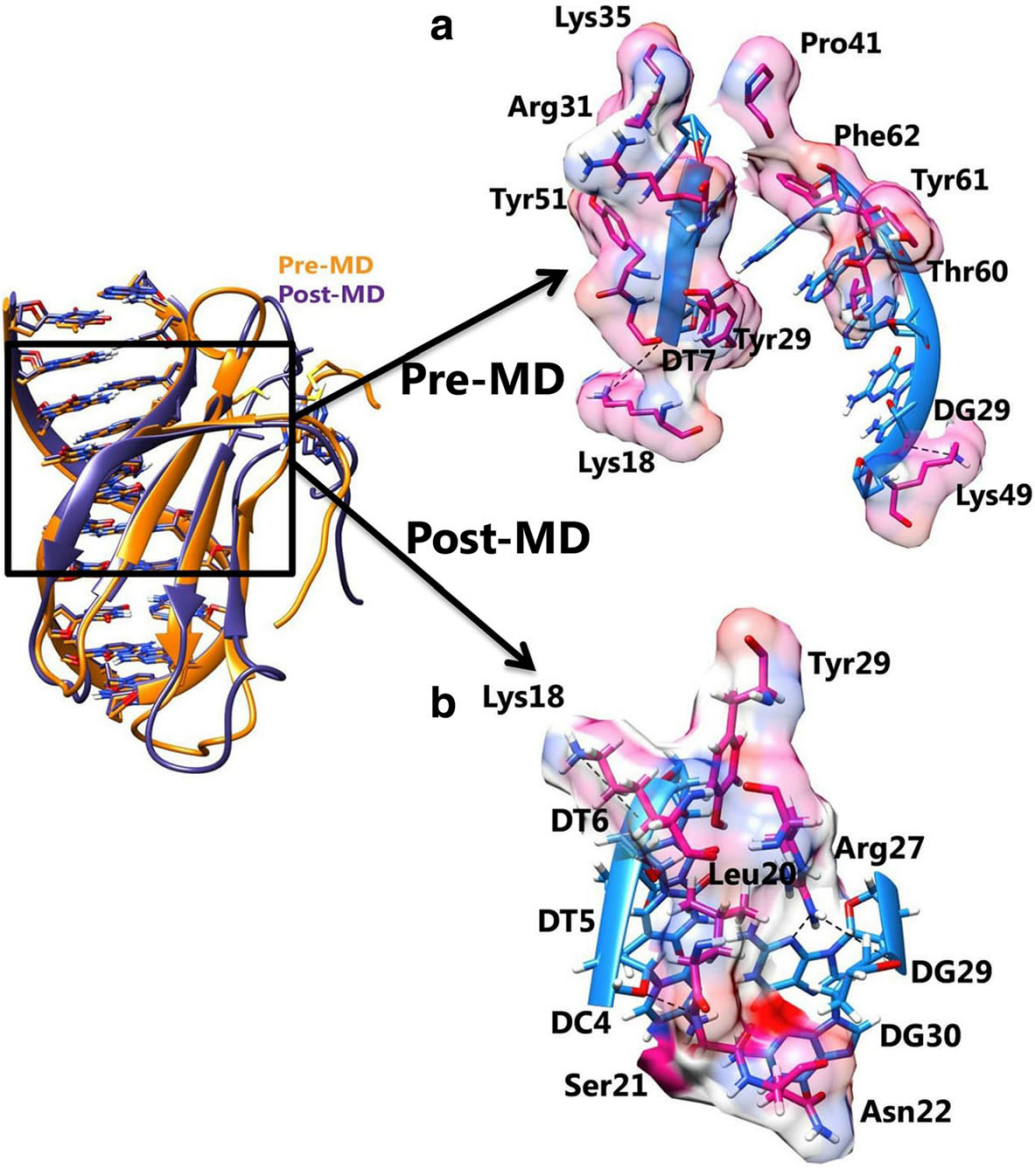
Interaction between Variant I WRKY DBD and DNA illustrating specific binding sites in (**a**) pre and (**b**) post-MD simulations

Cluster 4 and 2 for the variant I and variant II yielded highest HADDOCK score of − 126.2 ± 14.1 and − 118.5 ± 11.4 respectively and chosen for interaction analysis (Tables 6 and 7). For variant I, the complex was stabilized by four H-bonds and several hydrophobic interactions. H-bond was formed by Gly16, Lys18, Arg49 and Thr60 residues and residues involved in the formation of hydrophobic interaction were Tyr15, Ser21, Tyr29, Arg31, Lys35, Pro41, Thr43, Met51, Ser52, Thr58, and Tyr61 (Table 8; Fig. 6a).

**Table 6:** Protein-DNA complexes generated for Variant I WRKY DBD with DNA motif using HADDOCK server

Models	HADDOCK score (a.u)	RMSD (A°)	Van der Waals energy (Kcal/mol)	Electrostatic energy (J)	Desolvation energy (kcal/mol	Restraints violation energy(kcal/mol)	Buried Surface Area (Å^2^)	Z-Score
Cluster 1	−111.5 ± 3.5	14.2 ± 0.3	−56.4 ± 2.1	− 310.6 ± 10.1	5.1 ± 2.7	18.2 ± 12.32	1365.8 ± 31.7	−0.8
Cluster 2	−109.8 ± 3.7	13.9 ± 0.3	− 57.2 ± 3.3	− 303.9 ± 31.6	5.6 ± 2.1	25.2 ± 13.49	1478.0 ± 111.5	− 0.8
Cluster 3	− 96.9 ± 11.6	5.5 ± 0.6	− 40.7 ± 4.4	− 287.7 ± 53.2	−1.2 ± 3.3	25.4 ± 23.22	1179.2 ± 145.5	− 0.2
**Cluster 4**	**− 126.2 ± 14.1**	**1.2 ± 0.7**	**−57.6 ± 12.6**	**− 344.7 ± 34.8**	**− 10.6 ± 4.2**	**109.4 ± 17.57**	**1563.5 ± 150.0**	**−1.5**
Cluster 5	− 100.3 ± 7.4	14.8 ± 0.3	− 43.4 ± 8.5	−279.5 ± 5.2	− 8.9 ± 3.1	79.5 ± 44.82	1321.6 ± 73.7	−0.3
Cluster 6	−78.2 ± 10.1	13.3 ± 0.4	− 25.2 ± 5.3	− 288.4 ± 47.8	3.3 ± 6.0	13.7 ± 15.88	780.0 ± 88.8	0.7
Cluster 7	− 94.6 ± 11.6	14.0 ± 0.4	− 43.4 ± 6.9	− 260.7 ± 24.9	−12.7 ± 2.2	136.4 ± 29.71	1187.3 ± 189.7	− 0.1
Cluster 8	− 71.3 ± 7.8	15.5 ± 0.2	− 18.8 ± 3.0	− 311.7 ± 25.5	6.1 ± 4.6	37.3 ± 6.95	709.4 ± 25.7	1.0
Cluster 9	−48.5 ± 3.2	10.9 ± 0.6	−26.2 ± 2.9	−99.0 ± 17.5	−2.8 ± 4.6	2.8 ± 0.69	829.9 ± 102.4	2.0

Bold cluster selected for further analysis

**Table 7:** Protein-DNA complexes generated for Variant II WRKY DBD with DNA motif using HADDOCK server

Models	HADDOCK score (a.u)	RMSD (A°)	Van der Waals energy (Kcal/mol)	Electrostatic energy (J)	Desolvation energy (kcal/mol	Restraints violation energy(kcal/mol)	Buried Surface Area (Å^2^)	Z-Score
Cluster 1	−111.3 ± 11.2	7.5 ± 0.4	− 43.5 ± 8.1	−426.2 ± 24.8	15.0 ± 2.7	25.6 ± 17.83	1218.1 ± 124.8	− 1.0
**Cluster 2**	**− 118.5 ± 11.4**	**14.3 ± 0.2**	**− 53.0 ± 6.8**	**− 432.7 ± 53.8**	**18.0 ± 3.5**	**30.9 ± 16.03**	**1352.2 ± 99.5**	**− 1.3**
Cluster 3	−85.1 ± 4.7	13.0 ± 0.3	−25.0 ± 1.4	− 367.1 ± 25.7	12.8 ± 5.0	5.4 ± 5.40	807.4 ± 92.2	0.2
Cluster 4	−84.9 ± 1.8	8.0 ± 0.5	− 25.7 ± 2.3	− 381.0 ± 27.2	12.5 ± 4.1	44.6 ± 25.94	783.0 ± 61.6	0.2
Cluster 5	−88.6 ± 13.6	13.3 ± 0.2	− 28.5 ± 7.9	− 379.0 ± 52.9	12.6 ± 1.6	30.9 ± 16.98	979.1 ± 137.6	0.1
Cluster 6	−52.9 ± 15.3	8.0 ± 0.4	− 21.5 ± 4.0	− 245.1 ± 54.7	15.7 ± 4.7	19.1 ± 16.23	758.8 ± 93.4	1.8

Bold cluster selected for further analysis

**Table 8:** Residues involved in hydrogen and Hydrophobic interactions in the WRKY-DNA variants

WRKY variants	Pre-simulated docked complex		Post-simulated docked complex	
	Hydrogen bond	Hydrophobic interactions	Hydrogen bond	Hydrophobic interactions
Wild-type	Arg13-DA28 (2.9 Å), Gln17-DG8 (2.8 Å) Lys18-DT6 (2.9 Å, Lys18-DT7 (2.9 Å), Lys31-DG29 (2.7 Å), Arg40-DG30 (2.9 Å,2.9 Å)	Val19, Pro26	Arg13-DA29 (3.0 Å), Lys14-DA28(2.9 Å), Gln17-DA8(2.8 Å)	Asn25, Arg45, Lys51
Variant I	Gly16-DG8 (2.8 Å), Lys18-DT7 (2.8 Å), Arg49-DG30 (2.7 Å), Thr60-DA28 (2.9 Å)	Tyr15, Ser21, Tyr29, Arg31, Lys35, Pro41, Thr43, Met51, Ser52, Thr58, Tyr61	Lys18-DT6 (2.9 Å), Ser21-DC4 (2.8 Å), Arg27-DG29(2.9 Å), Arg27-DG30(2.7 Å)	Leu20, Asn22, Tyr29
Variant II	Lys18-DT7 (2.8 Å), Lys49-DG30 (2.7 Å)	Tyr15, Ser21, Tyr29, Arg31, Lys35, Pro41, Thr43, Met51, Ser52, Thr58, Tyr61	Ser21-DT7 (2.7 Å), Ser23-DG29 (2.7 Å), Asn24-DG30 (2.9 Å), Arg31-DA9 (2.8 Å, 2.8 Å)	Tyr15, Lys18, Asn22, Arg27, Lys35, Lys49

**Fig. 7:**
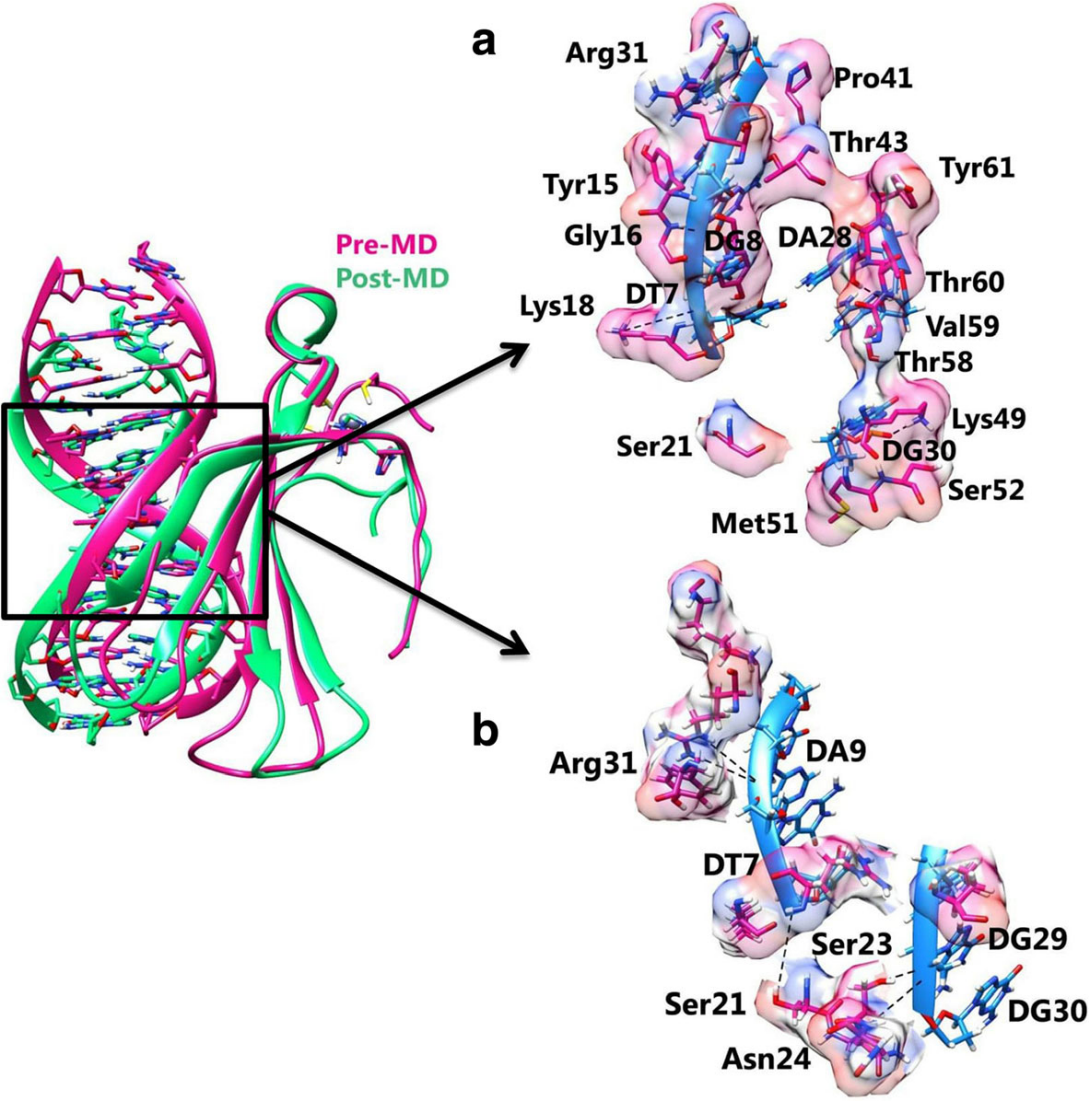
Interaction between Variant II WRKY DBD and DNA illustrating specific binding sites in (**a**) pre and (**b**) post-MD simulations

The detailed binding analysis of variant II cluster revealed the formation of two H-bonds by Lys18 and Lys49 (Table 8; Fig. 7a). The residues involved in hydrophobic interaction are listed in Table 8. Overall, the structural insight of WRKY domain-DNA complex revealed that hydrogen bond interactions play an essential role in stabilizing the protein-DNA complex along with the hydrophobic interactions.

### Molecular dynamics simulations of the docked complexes

**Fig. 8:**
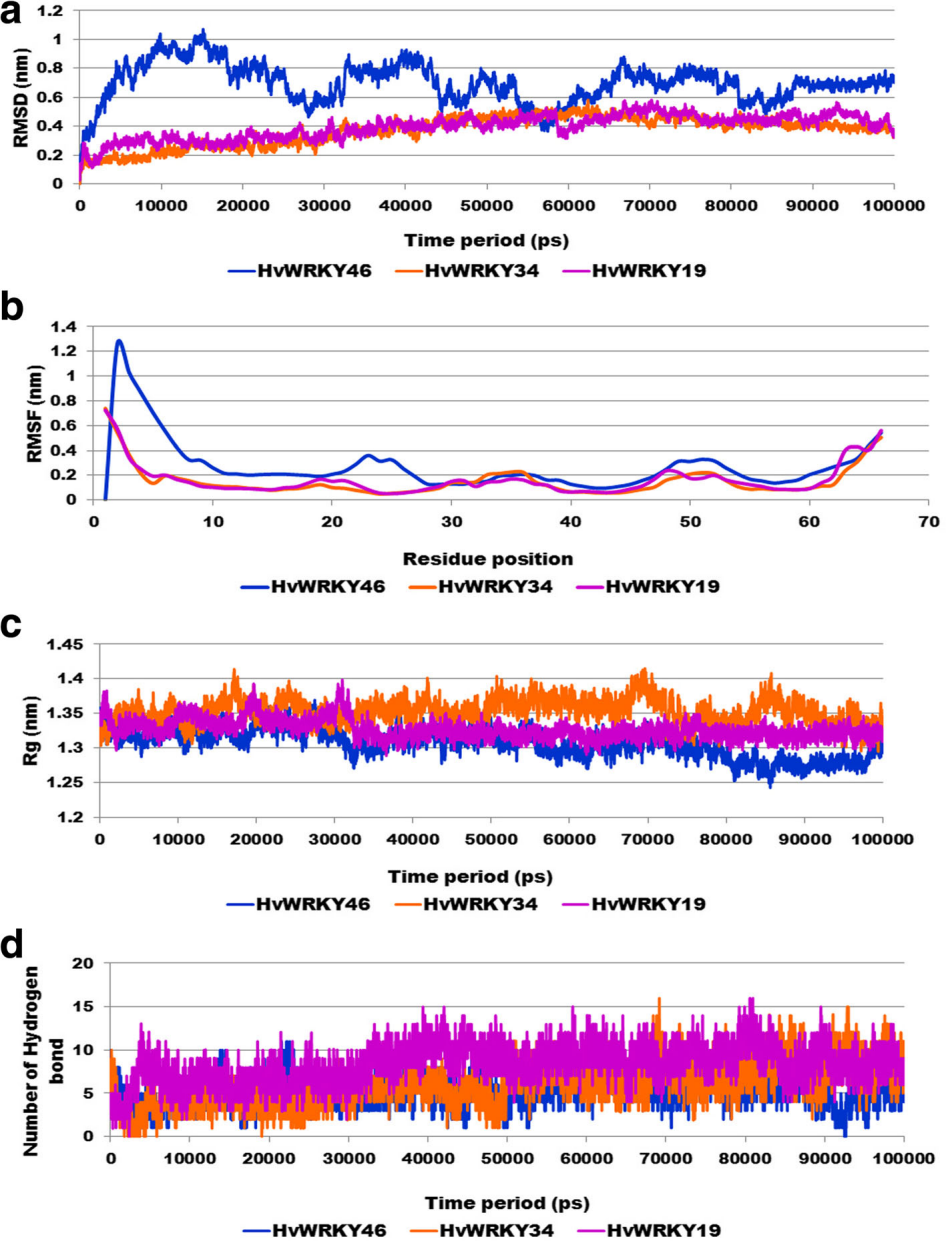
Graph showing the (**a**) RMSD plot (**b**) RMSF plot and (**c**) radius of gyration (Rg) (**d**) Number of hydrogen bond for wild-type and variants WRKY DBD bound with DNA

To examine the stability of protein-DNA complexes were subjected to long MD simulations of the 100 ns time period. The overall stability of each WRKY complex was measured by estimating the RMSD profile. RMSD deviation in case of wild-type-DNA complex was observed to be high as compared to variants (I and variant II) complexes. Moreover, wild-type attained overall complex stability after 90,000 ps simulation time period (Fig. 8a). From the RMSF plot, it was observed that heptapeptide residues of wild-type experienced slightly higher fluctuation than the variants, which enable wild-type to form stable interaction with the DNA during simulation as compared to variants (I and II) (Fig. 8b). Consequently, wild-type and variant II complexes were more compact (low Rg value) than variants I, resulted in the stable protein-DNA complex (Fig. 8c). Correspondingly, RMSF and Rg profiles for the wild-type were consistent with their resultant RMSD profiles.

Variation was observed in the number of hydrogen bonds formed between wild-type and variants (I and II) WRKY DBD and DNA motif during simulation time period (Fig. 8d). The wild-type and variants (I and II) exhibited H-bonds in the range from 5 to15 number during the entire simulation period. Total energy of the wild-type, variants (I and II) WRKY DBD-DNA complexes were found to be -546,668 kJ/mol, − 459,946 kJ/mol and -459,869 kJ/mol respectively, showed that higher energy corresponding to better stability of the wild-type WRKY DBD-DNA complex.

### Comparative interaction profile after molecular dynamics simulations

The simulated complex of WRKY-DNA was subjected to interaction pattern analysis to investigate main residues involved in H-bond and hydrophobic interactions. The wild-type WRKY-DNA complex was strongly stabilized by three H-bonds formed by Arg13, Lys14 and Gln17 residues and three residues (Asn25, Arg45 and Lys51) were involved in hydrophobic interactions. All residues were reported to be crucial for binding to DNA (Fig. 5b; Table 8) [42]. It is apparent from previously published WRKY protein-DNA NMR data [17] and MD simulations [18] from *Arabidopsis,* that more amino acids are necessary for the specific protein-DNA interaction of the WRKY DBD in addition to the conserved residue of the β1 strand.

For the variant I, the WRKY DBD and DNA complex was stabilized by H-bonds formed by Lys18, Ser21, Arg27, and Arg27 residues (Fig. 6b; Table 8). The variant II complex was stabilized by four H-bonds (Fig. 7b; Table 8). The difference in the hydrophobic interactions for the variants (I and II) was also noticed in the MD simulated complex (Table 8).

From the above result it was observed that substitution in the invariable WRKYGQK motif reduces the number of interaction and significant decline the DNA-binding activity and may even abolish the DNA-binding [36].

### MM-PBSA binding free energy calculations

Protein-DNA complexes were ranked based on protein binding affinity to DNA through MM-PBSA, a valuable binding free energy determining tool. MM/PBSA calculation was performed to calculate binding free energies for 14 different protein-DNA complexes in *Arabidopsis* from stable 5 ns of MD simulation trajectory, which revealed specific binding in AtWRKY1 cDBD–DNA complex [18]*.* Based on the 100 ns MD trajectories of wild-type and variants, binding free energy analysis and its corresponding components were analysed from the MM/PBSA calculation and reported in Table 9. The results indicated that wild-type exhibited a relatively high binding free energy value of -500.22 kJ/mol as comapred to variant I and II with a free energy value of -482.61 and -453.04 kJ/mol. Therefore, the point mutation decreased the positive polar term resulting in an overall increase of the negative term which promotes complex formation. The contribution of each residue towards binding energy was provided for wild-type, variant I and variant II respectively. It was observed in wild-type and variant II that the conserve heptapeptide (WRKYGQK) made major contribution whereas in variant I major contribution was made by Lys18 from heptapeptide [Additional file 5: Figure S5 (a-c); Additional file 6: Table S1; Additional file 7: Table S2; Additional file 8: Table S3]. Binding free energy components including van der Waals, electrostatic interaction, and non-polar solvation energy contribute negatively and favor complex formation. However, complex stability was majorly due to electrostatic interaction whereas apolar solvation energy contributes very less to the total energy of complex.

**Table 9:** Binding free energy calculation for WRKY DBD with DNA motif using MM/PBSA

Protein-DNA Complex	Van der Waals (kJ/mol) ΔGvdW	Electrostatic (kJ/mol) ΔGcoul	Polar contribution (kJ/mol) ΔGpolar	Non-polar contribution (kJ/mol) ΔGnonpolar	Free energy (kJ/mol) ΔG
Wild-type	−123.56	− 8221.15	8044.48	−200.00	−500.22
Variant I	−56.11	− 7907.15	7689.27	− 208.61	− 482.610
Variant II	− 346.46	− 7132.06	7206.29	-180.80	-453.04

### Principal component analysis

**Fig. 9:**
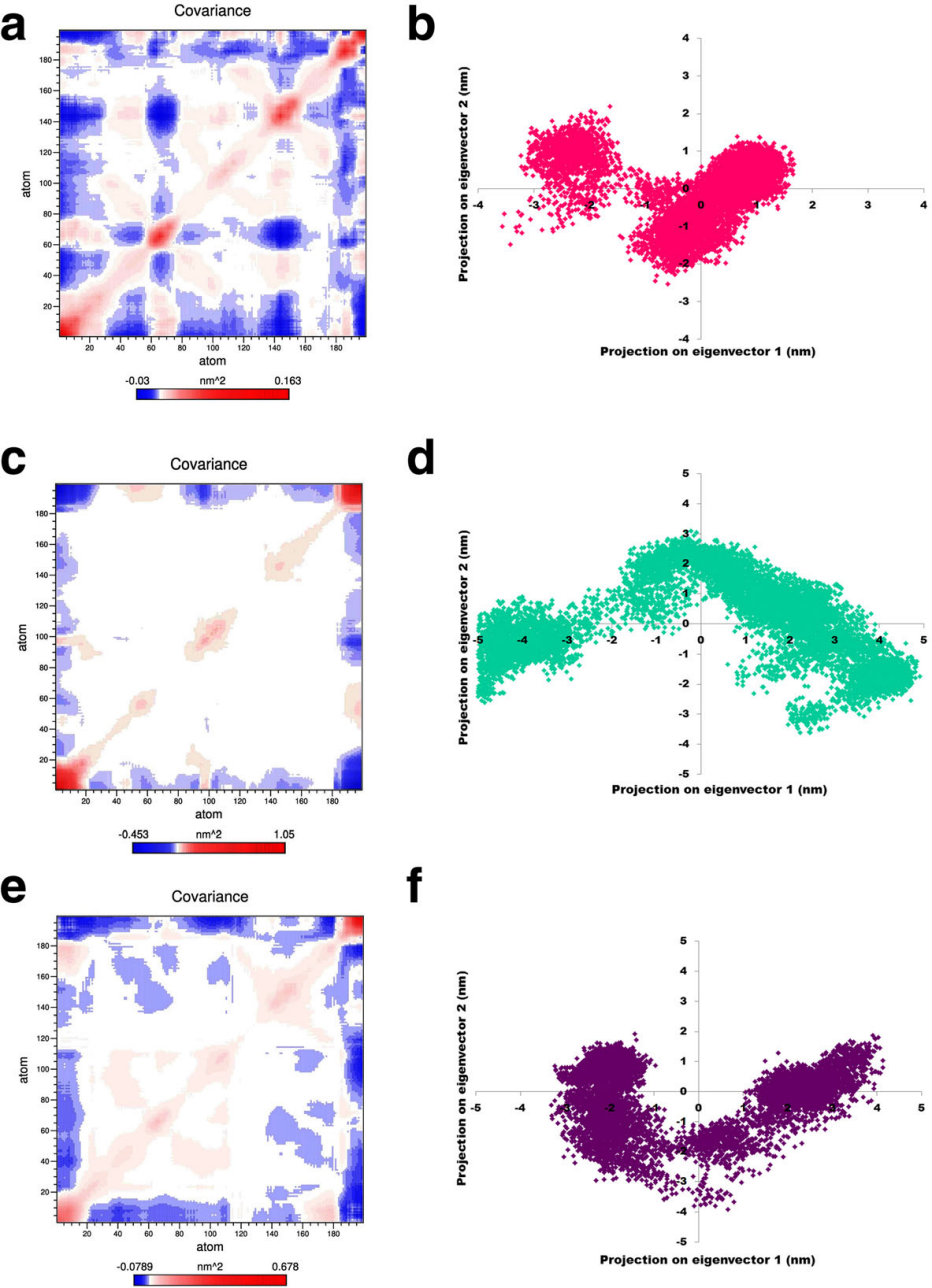
Computed covariance matrix for (**a**) wild-type (**c**) variant I (**e**) variant II. Red corresponds to a positive correlation shows the motion of the atom along the same direction and blue signifies the negative correlation indicates motion in opposite directions. Projection of the motion of the protein in phase space along the first two principal eigenvectors at 300 K was shown for (**b**) wild-type (**d**) variant I and (**f**) variant II

To better understand the structure and conformational changes, MD trajectories of the wild-type and variants (I and II) structures in DNA bound form were subjecetd to PCA analsyis. From the covariance plot, we depicted the positive and negative limits; positive values are related to the motion of the atoms occurring along the same direction whereas a negative value indicates motion of the atoms in the opposite direction. The highly anti-correlated motion was observed in wild-type WRKY complex as compared to variants (I and II) complexes [Fig. 9a–e].

The trace value for wild-type, variant I and variant II was found to be 4.1nm^2^ 15.9 nm^2^ and 8.61 nm^2^, respectively. High trace values for the variants indicated increased structural flexibility as compared to wild-type WRKY during the simulations time period. As a result of increased flexibility, conformational space covered by variants complexes was larger than the wild complex [Fig. 9b–f]. Thus, from above results, it was concluded that wild- type was more stable than the variants complex.

### Free energy landscape of wild-type and variants WRKY DBD

Gibbs free energy generates multi-dimensional free-energy plots, attributed to the area coverage by the data points in conformation space at 300 K [43]. In order to further analyze the PC projections, free energy landscapes for wild- type and variants were plotted. The structures from the start of simulation (0 ns) were on the right side and from the end of simulation time period (100 ns) were to the left side in each projection of PC. The free energy landscape analysis elucidated drastic conformational change in variants structure. The ∆G values for wild-type, variant I and variant II ranged from 14 kJ/mol, 12.9 kJ/mol and 15.6 kJ/mol respectively [Additional file 9: Figure S6 (A, B and C)].

## Conclusions

To better understand the structural basis for DNA recognition by the WRKY DBD, we integrated molecular and essential dynamics approach. Three-dimensional (3D) structures of WRKY domain from barley was built by homology modeling based on crystal structure of *Arabidopsis WRKY* gene. On 100 ns simulation trajectory, different tools were employed to examine the molecular behavior of wild and variants (Q17E and Q17K) complexes. Structural validations for all WRKY domains in unbound and bound form was done by RMSD, RMSF and Rg analysis. Based on RMSD and RMSF analysis, we confirmed that variant I showed higher deviation and fluctuation as compared to wild-type and variant II in unbound form. In order to investigate the effect of point mutations in structural and function of the protein, molecular docking was performed between WRKY DBDs and DNA for wild-type and variant complexes. Hydrogen bonds and hydrophobic interactions play a significant role in stabilizing the protein and DNA interaction. From MD simulation of 100 ns, we concluded that the mutation in the conserved amino acid residue in WRKY DNA binding domain has changed the WRKY protein natural behavior and interaction profile with DNA along with the stability of the complex. Taken together, we examined the distinct functional roles of conserved residues such as Trp12, Arg13, Lys14, Glu17, and Lys18 accentuating the mechanisms of DNA recognition for WRKY family which lead to regulation of their potential to defined target genes. Our outcome delivers efficiently new fundamental insights into the structural and thermodynamic geneses of protein-DNA binding specificity and thus has important implications for the prediction of transcription factor binding sites in genomes. It also offers that these mutations may have aberrant effects on the development of transgenic plants so should be avoided during development of developmental phenotypes of transgenic plants.

## Additional files

Additional file 1: **Figure S1.** BlastP analysis for the (a) HvWRKY46 (b) HvWRKY34 and (c) HvWRKY19. (EPS 44112 kb)

Additional file 2: **Figure S2.** Top five clusters were extracted from 100 ns MD simulations trajectory for (a) wild-type WRKY DBD (b) variant I and (c) variants II. (EPS 49108 kb)

Additional file 3: **Figure S3.** Secondary structure analysis for the (a) HvWRKY46 (b) HvWRKY34 and (c) HvWRKY19 using 100 ns MD simulation trajectories. (EPS 20756 kb)

Additional file 4: **Figure S4.** Electrostatic potential molecular surfaces for (a) WRKY protein (b) WRKY-DNA complex. Generally the red symbolizes negative charge and blue positive. (EPS 30224 kb)

Additional file 5: **Figure S5.** Per residue contriution towards binding free energy was calculated for (a) wild-type WRKY DBD (b) variant I (c) variant II. (EPS 42533 kb)

Additional file 6: **Table S1.** Per residue calculation was performed for wild-type. (PDF 89 kb)

Additional file 7: **Table S2.** Per residue calculation was performed for variant I. (PDF 89 kb)

Additional file 8: **Table S3.** Per residue calculation was performed for variant II. (PDF 89 kb)

Additional file 9: **Figure S6.** Illustrating gibbs free energy for (a) Wild WRKY (b) variant I (c) variant II. (EPS 49782 kb)

## Abbreviations

DA: Deoxyadenosine
DC: Deoxycytidine
DG: Deoxyguanosine
DT: Deoxythymidine
Rg: Radius of gyration
RMSD: Root-mean-square deviation
RMSF: Root mean square fluctuation
SASA: Solvent-accessible surface area
