# Supplementary material for: Molecular dynamics simulations revealed structural differences among WRKY domain-DNA interaction in barley (Hordeum vulgare)
Source: BMC Genomics. 2018 Feb 12;19:132. doi: 10.1186/s12864-018-4506-3 (PMC5810047; doi:10.1186/s12864-018-4506-3)
Supplement: Supplementary file 6 — Table S1. Per residue calculation was performed for wild-type. (PDF 89 kb) [file 12864_2018_4506_MOESM6_ESM.pdf]

Table S1: Per residue calculation was performed for wild-type.

#Residues	MM	Polar	APolar
SER-1	-438.168	1.3491	-0.0053
GLU-2	458.7138	0.8689	-0.001
VAL-3	-0.5102	-0.0919	0.0006
ASP-4	429.5654	0.8608	-0.0013
ILE-5	-10.4061	0.0874	0
LEU-6	8.6173	0.7183	0.0017
ASP-7	542.9541	0.1761	-0.0007
ASP-8	515.748	-0.6331	0.0014
GLY-9	-0.1166	-0.4698	-0.0001
TYR-10	9.7911	0.6282	0.0008
ARG-11	-624.315	6.8463	-0.0093
TRP-12	19.0593	-3.4657	-0.1115
ARG-13	-972.507	140.0335	-1.8505
LYS-14	-912.272	70.7479	-1.2104
TYR-15	-3.0978	2.4494	-0.2062
GLY-16	1.2721	-1.9976	-0.0031
GLN-17	0.5618	10.37	-0.8556
LYS-18	-537.117	5.2431	-0.0031
VAL-19	-3.4774	-0.1248	0.003
VAL-20	6.4259	0.2736	-0.0033
LYS-21	-411.891	1.1237	0.0001
GLY-22	0.4492	0.3367	0.0005
ASN-23	5.0811	0.4033	-0.0052
PRO-24	9.9226	0.1908	0.0014
ASN-25	0.7829	0.9247	-0.0054
PRO-26	-2.9854	0.9594	-0.1459
ARG-27	-524.45	10.1062	-0.002
SER-28	-15.3704	2.0989	-0.0534
TYR-29	25.4215	-3.5327	0.0066
TYR-30	-29.3234	5.3967	-0.0611
LYS-31	-603.789	20.5356	-0.0107
CYS-32	-6.8616	0.5018	0.0104
THR-33	-12.3518	1.6184	0.0027
SER-34	6.9982	-1.1718	0.0033
THR-35	2.2888	0.3496	0.0013
GLY-36	-5.0979	0.3438	-0.0024
CYS-37	6.4448	-0.728	0.0089
PRO-38	6.5926	-0.324	0.0022
VAL-39	13.2184	-1.3127	-0.0013
ARG-40	-529.138	10.8344	-0.0055
LYS-41	-618.08	12.9799	0.0014

HIS-42	-8.4261	0.8033	0.0029
VAL-43	4.4907	-0.9476	-0.0016
GLU-44	535.3162	-9.5847	-0.0028
ARG-45	-916.656	105.0201	-1.3046
ALA-46	14.6696	-0.4068	0.0014
SER-47	9.4557	0.6348	-0.0014
HIS-48	8.8687	1.1448	0.0007
ASP-49	495.5588	-3.3173	-0.006
PRO-50	-14.1827	1.0554	-0.0081
LYS-51	-816.003	73.7785	-0.8168
SER-52	10.6469	-0.9574	0.0006
VAL-53	-10.534	1.048	-0.0104
ILE-54	12.013	-0.9195	0.0045
THR-55	-0.531	0.7511	0.0015
THR-56	3.6332	-0.4634	0.0049
TYR-57	-2.0187	0.6754	0.0047
GLU-58	482.1997	-6.6574	0.0005
GLY-59	5.2465	-0.4824	0.0049
LYS-60	-351.556	0.9124	0.0075
HIS-61	-14.8892	0.5636	0.0021
ASN-62	13.0377	-0.3426	0.0074
HIS-63	13.525	-0.8223	-0.0018
GLU-64	378.801	-0.3589	-0.0005
VAL-65	-3.0783	0.3345	-0.0007
PRO-66	443.1103	0.2946	-0.0041
