# Supplementary material for: Molecular dynamics simulations revealed structural differences among WRKY domain-DNA interaction in barley (Hordeum vulgare)
Source: BMC Genomics. 2018 Feb 12;19:132. doi: 10.1186/s12864-018-4506-3 (PMC5810047; doi:10.1186/s12864-018-4506-3)
Supplement: Supplementary file 7 — Table S2. Per residue calculation was performed for variant I. (PDF 89 kb) [file 12864_2018_4506_MOESM7_ESM.pdf]

Table S2: Per residue calculation was performed for variant I

#Residues	MM	Polar	APolar
ALA-4	-606.37	13.3241	-0.0275
PRO-5	7.0342	-1.2519	-0.0006
TYR-6	3.9648	-0.991	-0.0014
ASP-7	488.878	-9.4263	0.0015
ASP-8	566.654	-23.461	0.001
GLY-9	-3.2491	0.4188	-0.0024
HIS-10	-18.76	2.2896	-0.0027
GLN-11	13.2216	-2.4179	0.0041
TRP-12	-7.5487	1.2232	-0.0006
ARG-13	-549.81	14.393	-0.0047
LYS-14	-565	22.8299	-0.0006
TYR-15	-32.121	11.2881	-0.5714
GLY-16	1.3839	-1.7113	-0.0012
GLU-17	565.67	-19.024	0.0003
LYS-18	-933.74	136.437	-1.2022
LYS-19	-634.36	34.982	-0.0354
LEU-20	-51.132	13.3316	-0.9375
SER-21	-35.935	20.4687	-1.2326
ASN-22	-29.506	18.2338	-1.2398
SER-23	-40.124	21.1214	-0.9232
ASN-24	-64.729	22.5927	-0.4265
PHE-25	13.9397	-3.1544	-0.2907
PRO-26	6.7218	-2.2823	0.0017
ARG-27	-935.15	148.32	-1.367
PHE-28	13.1564	-3.7863	0.0027
TYR-29	-34.87	12.7641	-1.0305
TYR-30	27.1209	-5.7583	-0.0082
ARG-31	-898.02	108.564	-0.7328
CYS-32	0.9144	0.4557	0.0006
THR-33	7.9741	-0.882	0.0025
TYR-34	-3.0185	0.9624	0.0073
LYS-35	-902.25	124.664	-0.7222
THR-36	3.3686	-0.9215	-0.0096
ASP-37	500.995	-6.3127	0.0063
LEU-38	-2.1044	0.6936	-0.0026
LYS-39	-630.39	15.6616	-0.0968
CYS-40	6.8422	-2.9409	0.0011
PRO-41	20.2239	-4.1696	-0.3347

ALA-42	1.209	-1.8672	0.0098
THR-43	-7.4074	5.869	-0.8539
LYS-44	-600.02	31.8185	-0.0034
GLN-45	-13.189	3.6836	-0.4153
VAL-46	7.496	-1.9675	0.0041
GLN-47	-17.844	12.9594	-0.7066
GLN-48	24.1798	-5.0244	0.0148
LYS-49	-773.26	87.0996	-0.4781
ASP-50	517.527	-15.786	0.0045
MET-51	-15.567	1.9834	-0.0131
SER-52	-10.466	1.0944	-0.0015
ASP-53	448.702	-6.6479	0.0016
PRO-54	10.4112	-1.2286	-0.0006
PRO-55	5.9527	-0.6618	0.0069
LEU-56	1.8947	-0.9778	0.0007
PHE-57	-13.585	3.8508	0.0113
THR-58	-6.1455	-1.3352	-0.0725
VAL-59	4.4139	0.2613	-0.0108
THR-60	-23.679	9.0591	-1.008
TYR-61	12.9983	-0.9664	-0.2652
PHE-62	-30.922	7.2892	-1.7111
ASN-63	-25.009	12.548	-0.9076
HIS-64	19.9387	-0.6257	-0.0056
HIS-65	9.8106	-2.2019	-0.0002
SER-66	-3.869	-0.8249	-0.0057
CYS-67	-7.0258	0.61	0.0015
ASN-68	2.6957	-0.7218	0.0102
THR-69	602.118	-12.334	-0.0462
