# Supplementary material for: Molecular dynamics simulations revealed structural differences among WRKY domain-DNA interaction in barley (Hordeum vulgare)
Source: BMC Genomics. 2018 Feb 12;19:132. doi: 10.1186/s12864-018-4506-3 (PMC5810047; doi:10.1186/s12864-018-4506-3)
Supplement: Supplementary file 8 — Table S3. Per residue calculation was performed for variant II. (PDF 89 kb) [file 12864_2018_4506_MOESM8_ESM.pdf]

Table S3: Per residue calculation was performed for variant II

#Residues	MM	Polar	APolar
ALA-4	-606.397	13.3319	-0.0275
PRO-5	7.0513	-1.2464	-0.0008
TYR-6	3.9681	-0.9925	-0.0016
ASP-7	488.9425	-9.4305	0.0013
ASP-8	566.63	-23.4544	0.001
GLY-9	-3.2487	0.4185	-0.0025
HIS-10	-18.7517	2.2937	-0.0027
GLN-11	13.2178	-2.421	0.0043
TRP-12	-7.5488	1.2247	-0.0004
ARG-13	-549.752	14.3947	-0.0047
LYS-14	-564.997	22.8333	-0.0007
TYR-15	-32.1288	11.2998	-0.5713
GLY-16	1.3691	-1.7132	-0.0012
GLU-17	565.6578	-19.0217	0.0005
LYS-18	-933.932	136.4686	-1.2017
LYS-19	-634.398	35.0199	-0.035
LEU-20	-51.115	13.3297	-0.9379
SER-21	-35.986	20.4516	-1.2326
ASN-22	-29.4732	18.2159	-1.2403
SER-23	-40.1313	21.1348	-0.9233
ASN-24	-64.7465	22.5863	-0.4257
PHE-25	13.9357	-3.1518	-0.2908
PRO-26	6.7283	-2.2855	0.0016
ARG-27	-935.151	148.3096	-1.3675
PHE-28	13.1531	-3.7871	0.0028
TYR-29	-34.863	12.7605	-1.0305
TYR-30	27.1254	-5.7557	-0.0083
ARG-31	-897.996	108.5941	-0.7332
CYS-32	0.9107	0.4591	0.0006
THR-33	7.9584	-0.8802	0.0028
TYR-34	-3.0243	0.9599	0.0076
LYS-35	-902.262	124.6679	-0.7216
THR-36	3.3685	-0.9188	-0.0094
ASP-37	501.0049	-6.3113	0.0061
LEU-38	-2.1021	0.6934	-0.0026
LYS-39	-630.532	15.612	-0.0966
CYS-40	6.842	-2.9364	0.0015
PRO-41	20.2038	-4.1652	-0.3348

ALA-42	1.2029	-1.8686	0.0098
THR-43	-7.3842	5.8714	-0.8535
LYS-44	-600.017	31.8208	-0.0035
GLN-45	-13.1961	3.6834	-0.4154
VAL-46	7.5017	-1.9701	0.0041
GLN-47	-17.8811	12.9562	-0.706
GLN-48	24.1656	-5.0163	0.0148
LYS-49	-773.257	87.0548	-0.4789
ASP-50	517.4935	-15.7877	0.0046
MET-51	-15.5743	1.9822	-0.0131
SER-52	-10.4764	1.0927	-0.0016
ASP-53	448.7012	-6.6451	0.0017
PRO-54	10.4152	-1.2269	-0.0007
PRO-55	5.9599	-0.6622	0.0071
LEU-56	1.8911	-0.9783	0.0008
PHE-57	-13.5803	3.8496	0.0113
THR-58	-6.1484	-1.3331	-0.0724
VAL-59	4.4169	0.2625	-0.0108
THR-60	-23.6886	9.0713	-1.0082
TYR-61	12.9907	-0.9696	-0.2648
PHE-62	-30.925	7.2835	-1.7099
ASN-63	-25.0069	12.5655	-0.9084
HIS-64	19.9483	-0.6273	-0.0058
HIS-65	9.8014	-2.2037	-0.0001
SER-66	-3.8894	-0.8237	-0.0058
CYS-67	-7.03	0.6096	0.0013
ASN-68	2.7089	-0.7209	0.0103
THR-69	602.2365	-12.3408	-0.0462
